# The antigenic determinant that defines thymic nurse cells is expressed by thymic epithelial progenitor cells

**DOI:** 10.3389/fcell.2014.00013

**Published:** 2014-04-28

**Authors:** Rajendra V. E. Chilukuri, Viral K. Patel, Marcia Martinez, Jerry C. Guyden, Michael D. Samms

**Affiliations:** ^1^Department of Biology, The City College of New YorkNew York, NY, USA; ^2^Biology, Tuskegee UniversityTuskegee, AL, USA

**Keywords:** thymic nurse cells, K5^+^K8^+^, Foxn1, p63, thymic progenitor cells, mTEC and cTEC

## Abstract

Stromal thymic epithelial cells with the multicellular structure unique to thymic nurse cells (TNCs) express the pH91 antigen on their cell surfaces. The multicellular TNC-complexes develop through an intimate association between αβTCR^+^CD4^+^CD8^+^ thymocytes and pH91-expressing cortical epithelial cells. TNCs participate in MHC-restriction and exhibit epithelial cell progenitor characteristics. In this report, we show that as early as E11.5 stage of thymus development, the pH91 antigen is expressed in association with K8, K5, Foxn1, and p63. The expression of these epithelial progenitor markers along with the pH91 antigen persists throughout thymic development in the murine thymus. At E13.5, pH91^+^ cells express relatively low levels of MHC class II. After E17.5, the first multicellular TNC complexes are recognizable along with increased cell surface expression of MHC class II. Our data suggest that epithelial cells bearing the “progenitor phenotype” develop into the multicellular TNCs.

## Introduction

The bipotent population of cells that give rise to both cortical (cTECs) as well as medullary epithelial (mTECs) cells that participate in the maintenance of self-tolerance within the organism (Gill et al., 2002; Baik et al., 2013) has received much attention in recent years. Previous studies showed thymic epithelium (TECs) to be generated exclusively from the endoderm (Le Douarin and Jotereau, 1975; Gordon et al., 2004). By embryonic day 12, E12, the structural morphology of the thymus becomes visible, with the initial separation of the cortex and the medulla (Klug et al., 1998, 2002). This morphology results in a visibly densely packed cortex surrounding a markedly more defused medulla. The cells that comprise the cortex or the medulla can be distinguished by their cytoplasmic keratin profiles (Klug et al., 1998). Cells of the cortex express cytokeratin 8 (K8) but not cytokeratin 5 (K5), while the inverse expression pattern was found for cells of the medulla (Klug et al., 1998). This keratin expression pattern is preceded in the embryological thymus by cells that express both K8 and K5 (Gill et al., 2002; Klug et al., 2002). K8^+^K5^+^ cells were shown to appear between E11 and E12 (Gill et al., 2002; Klug et al., 2002). Since double positive epithelial cells appear before K8^+^K5^−^ or K8^−^K5^+^ single positives, it has been suggested that K8^+^K5^+^ cells are progenitors of the both single positive subsets, and give rise to cells that develop into the medulla or cortex. This theory is supported by studies in which T cell development is blocked early during the differentiation process (Klug et al., 1998). Such genetic mutations produce an “immature” thymus, which produce a stromal epithelial population that is predominantly K8^+^K5^+^ cells.

Besides cytokeratin characterization, other studies are providing important information regarding the common bipotent cell population that result in both cTECs as well as mTECs (Baik et al., 2013; Alves et al., 2014). The findings of one study showed that TEC progenitors undergo an orchestrated sequence of events leading to the concomitant expression of the cTEC identifier CD205 and also the Receptor Activator of NF-kB (RANK), the mTECs regulator. The DC205^+^ cells were shown to develop and produce functionally capable microenvironments consisting of cTECs and mTECs expressing the autoimmune regulator Aire (Baik et al., 2013; Alves et al., 2014). Another study employed the B5t-Cre-loxP mediated GFP expression system strongly support the notion that Aire-expressing medullary epithelial cells arise from B5t producing progenitor cells (Ohigashi et al., 2013). Further, other evidence suggest that Aire+ mTECs express the tight-junction components claudin-3 and claudin-4 early in thymus ontogeny (Hamazaki et al., 2007). These results suggest that heterogeneous populations of progenitor cells may differentiate into cTECs and/or mTECs.

The expression of transcription factors during early thymic organogenesis has provided some insights into the characterization of thymic epithelial progenitors (Manley, 2000; Gordon et al., 2001). Foxn1 has a winged-helix/forkhead DNA binding domain as well as a transcriptional activation domain (Biggs et al., 1999; Kops et al., 1999), and is the earliest marker known to indicate the commitment to the epithelial development of the thymus (Manley, 2000; Gordon et al., 2001). Foxn1 mutants have been shown to block the expansion of TEC, and resemble mutant mice with a block in thymocyte development, in that the epithelial development does not go beyond the K8^+^K5^+^ phenotype (Nehls et al., 1996). Another transcription factor, p63, an homolog of p53 is important in the maintenance of the proliferative potential of epithelial cells (Bleul et al., 2006; Gillard and Farr, 2006; Senoo et al., 2007). Its expression is up-regulated in putative TEC stem cells (Gillard and Farr, 2006). Other reports suggest that p63 functions in the commitment of the cells to the epithelial linage in the thymus (Blanpain and Fuchs, 2007; Candi et al., 2007). The expression of p63 is detectable by E12 within K8^+^K5^+^ cells in the developing thymus (Senoo et al., 2007).

In a previous report (Hendrix et al., 2010), we showed cells with the morphology of thymic nurse cells (TNCs) to express the TNC-antigen, pH91, K8, and K5 along with the transcription factor p63 (Hendrix et al., 2010). The pH91 antigen has been previously shown to participate in TNC binding and internalization of immature thymocytes at the triple positive stage of development (Pezzano et al., 1998). Further, blocking studies showed this antigen to be vital to the survival of the developing thymus (Pezzano et al., 1998). Although pH91 is an orphan antigen, its role during MHC restriction has been shown to be important to the development of the thymus (Pezzano et al., 1996; Samms et al., 1999; Martinez et al., 2007).

The ontogenic expression of pH91 antigen in conjunction with Foxn1 has not been previously investigated. In this report, we show the K8^+^K5^+^p63^+^ TNC-subset to express the transcription factor, Foxn1. Further, we show the onset of pH91 expression to be concomitant with that of Foxn1 within the third pharyngeal pouch on E11.5 day of development. A subset of cells with the pH91^+^ Foxn1^+^ phenotype persists throughout ontogeny and during the early neonatal stage of development. These data suggest that a subset of cells previously defined as thymic epithelial progenitors express the TNC-antigen, pH91.

## Materials and methods

### Isolation of TNCs and thymocytes

Isolation of cells were performed as previously described (Hendrix et al., 2010). Briefly, C57BL/6 mice (Jackson Laboratory, Bar Harbor, ME) were dissected aseptically and the thymi were removed. Thymi were slightly disrupted with fine needles and subjected to enzymatic digestion in a solution of 0.015% collagenase D (Sigma Aldrich, St Louis, MO), 0.01% DNAse I (Sigma Aldrich), and 25 ml of trypsin (GIBCO, Carlsbad, CA) along with gentle agitation. The solution was changed every 10 min until the thymi were completely digested. The resulting cells were subjected to 1×g gradient separation in fetal bovine serum (Atlas Biological, Fort Collins, CO) at 4°C to enrich TNC numbers. Thymocytes were obtained by the mechanical disruption of thymi obtained from 4 to 6 week old C57BL/6 mice. Macrophage depletion was accomplished by negative sorting using CD11b Microbeads (Miltenyi Biotech, Auburn, CA).

### Timed pregnancy and thymic sections

Experiments were conducted as previously described (Hendrix et al., 2010). C57BL/6 mice were mated overnight; females were separated the next day upon detection of a vaginal plug. For early timed embryo (E11.5 and E12.5 sections) the entire fetus was embedded in OTC medium. Form E13.5 onwards, individual lobes were isolated and embedded in OTC medium (Richard Allan Scientific, Kalamazoo, MI). Thymic sections, 10 μm in thickness were made using a Leica CM1950 Cryostat. Sections were mounted onto Bond-Rite microscope slides (Richard Allan Scientific, MI) for immunostaining and sections were analyzed using a Zeiss LSM510 Confocal Microscope. Phenotypic subsets of embryonic cells obtained from E11.5 and E12.5 were microscopically enumerated; more than 550 cells were counted for each embryonic time point.

### Immunostaining of TNCs and thymic sections

Experiments were conducted as previously described (Hendrix et al., 2010). Briefly, isolated TNCs were deposited onto glass slides using a Thermo Scientific Shandon Cytospin 4. Thymic sections or isolated TNCs were fixed in 2% paraformaldehyde (Baker, Phillipsburg, PA) for 30 min followed by 3 washes with PBS (GIBCO). Sections were blocked and permeabilized in 3% BSA (Fisher Scientific, Pittsburg, PA), 0.1% Triton-X (Fisher Scientific) in PBS. Samples were incubated with primary and secondary antibodies at 37°C for 1 h each. Samples were mounted in ProLongGold antifade with DAPI (Molecular Probes, Carlsbad, CA). Images were acquired using the Zeiss LSM510 Confocal Microscope. Primary antibodies used were as follows: rat anti-mouse pH91 monoclonal antibody (IgG2a), K8 TROMA-I (IgG2a) (Developmental Studies Hybridoma Bank, Iowa City, IA), chicken anti-mouse K8 polyclonal antibody (IgY) (Abcam, Cambridge, MA), goat anti-rabbit K5 polyclonal antibody PRB-160B (IgG) (Covance, Princeton, NJ), rabbit anti-goat Δ Np63 (N-16): sc-8609 (Santa Cruz Biotechnology, Santa Cruz, CA), rabbit anti-Foxn1 polyclonal antibody (IgG) H-270 (Santa Cruz Biotechnology), antibody FITC-conjugated anti-mouse MHC class II (Miltenyi Biotech), biotinylated anti-mouse αβ TCR (BD Pharmingen, San Jose, CA), APC-conjugated CD4 (BD Pharmingen), PE-conjugated CD8 (BD Pharmingen), FITC-conjugated Thy 1.2 (BD Pharmingen), FITC-conjugated rat IgG2a isotype control (BD Pharmingen), and TRITC-conjugated rabbit IgG2a isotype control (BD Pharmingen). Secondary antibodies used are as follows: FITC-conjugated mouse anti-rat IgG2a (BD Pharmingen), APC-conjugated donkey anti-chicken IgY (Jackson ImmunoResearch Laboratories, West Grove, PA), TRITC-conjugated goat anti-rabbit IgG (Jackson ImmunoResearch Laboratories), APC-conjugated donkey anti-rabbit IgG (Jackson ImmunoResearch Laboratories), TRITC-conjugated donkey anti-rabbit IgG (Jackson ImmunoResearch Laboratories), TRITC-conjugated donkey anti-goat IgG (Jackson ImmunoResearch Laboratories), TRITC-conjugated rabbit anti-goat IgG (Jackson ImmunoResearch Laboratories), and TRITC-conjugated streptavidin (BD Pharmingen).

### FACS analysis of isolated TNCs

Pregnant mice were euthanized and embryonic pups removed at E13.5 or E16.5 or E18.5 stages of development. Thymi from each embryonic stage were subjected to enzymatic digestion. Isolated cells were washed in ice cold EDTA/FACS buffer. Then, 5 × 10^5^ cells were then co-stained with pH91 mAb and anti-MHC II primary antibodies in 30 μl residual volume of EDTA/FACS buffer for 30 min at 4°C. Preparations were washed 3 times with 500 μl of buffer and incubated with secondary reagents in residual volume. Finally, cells were washed 3 times and resuspended in a 300 μl volume of EDTA/FACS buffer for analysis using a BD FACS analyzer.

### Statistical test

The student two-tailed *t*-test was used in the statistical analysis of data.

## Results

### TNCs express progenitor phenotype

**Figure 1:**
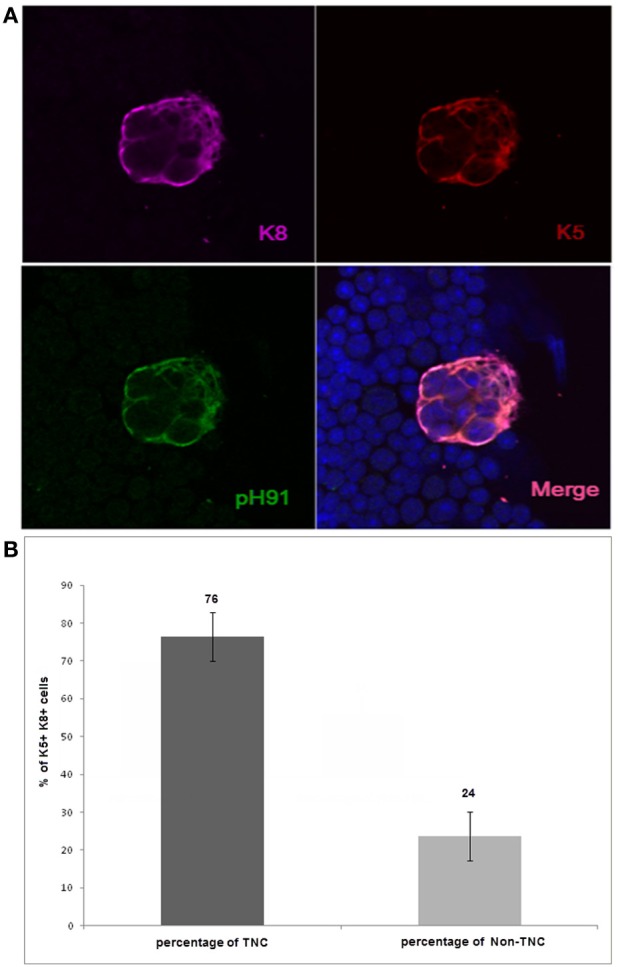
**Confocal analysis of K5^+^K8^+^pH91^+^*ex vivo* multicellular TNCs. (A)** shows isolated TNCs stained with anti-K8 antibody (magenta); anti-K5 antibody (red); and the TNC-specific antibody pH91 (green). **(A)** (lower right) shows a merged image. Original magnification 40×. **(B)** shows the quantification of the K5^+^K8^+^ pH91^+^TNCs as well as K5^+^K8^+^ non-TNCs. Isolated K5^+^K8^+^ cells were manually counted (1000) using a Zeiss confocal microscope. Data represent three independent expriments.

Thymic epithelial cells within the cortico-medullary junction (CMJ) that express both K5 and K8 have been proposed to be thymic epithelial progenitor cells (Klug et al., 2002). In a recent publication, we showed in tissue section that K8 and K5 positive TNCs reside within the CMJ of the thymus (Hendrix et al., 2010). To determine the frequency of these cytokeratin subsets, we isolated TNCs and co-stained with antibodies to K8 and K5 as well as the pH91-antigen. The results show that 76% of the epithelial cell population expressed both K8 and K5 and portrayed the multicellular morphology of TNCs, whereas 24% of the K5^+^K8^+^ cells did not exhibit the distinctive multicellular TNC phenotype (Figures 1A,B). Cells with the multicellular structures of TNCs that expressed the K5 only phenotype were not detected (data not shown).

### Expression of Foxn1 and pH91-antigen in thymic primordium

**Figure 2:**
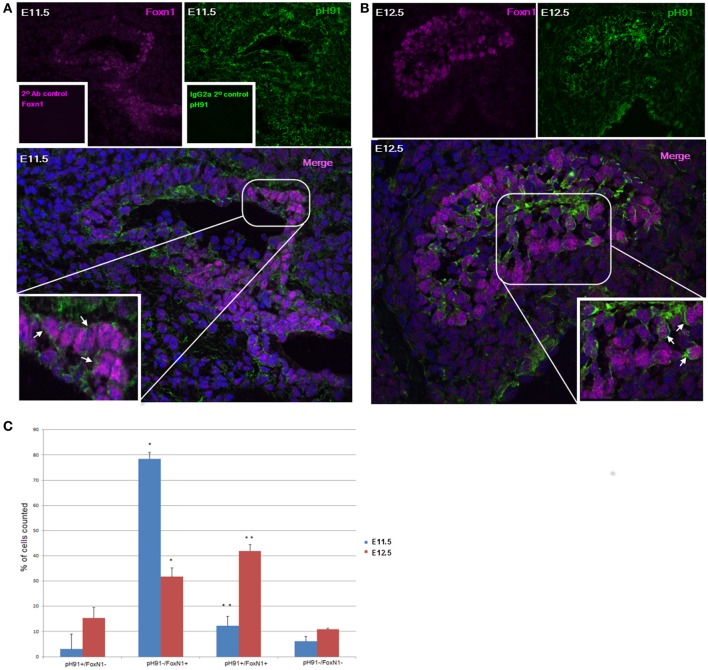
**Thymic embryonic sections stained with pH91 and anti-Foxn1 antibody. (A)** shows embryonic staining with anti-Foxn1 antibody (magenta), left panel; along with pH91 (green) right panel of E11.5 mouse thymic primordium. Expression of Foxn1 is localized toward the ventral region of the embryo whereas pH91 expression is seen throughout the entire thymic and para-thyroid primordium. The merged image (lower panel) and inset (enlarged) show pH91^+^ cells in association with Foxn1^+^ cells (arrows). The insets of the upper left (inset) and upper right (inset) panels show secondary antibody control for Foxn1 antibody (magenta) and IgG2a isotope control for pH91 (green). **(B)** shows staining of E12.5 thymic anlagen with anti-Foxn1 antibody, left panel (magenta) and pH91, right panel (green). The merged image (lower panel) and inset (enlarged) show an association of Foxn1^+^ with pH91^+^ cells (arrows). Magnification 40×. **(C)** shows the relative percentage of the various phenotypic subtypes present at E11.5 and E12.5 respectively. The pH91^−^/Foxn1^+^ phenotype shows a significant increase (^*^P < 0.001) between E11.5 and E12.5, (*n* = 6). The pH91^+^/Foxn1^+^ subset show statistical significance (^**^P < 0.05) between E11.5 and E12.5 (*n* = 6). Five-hundred and fifty embryonic cells were counted for **(C)**. The images are representative of three independent expriments.

**Figure 3:**
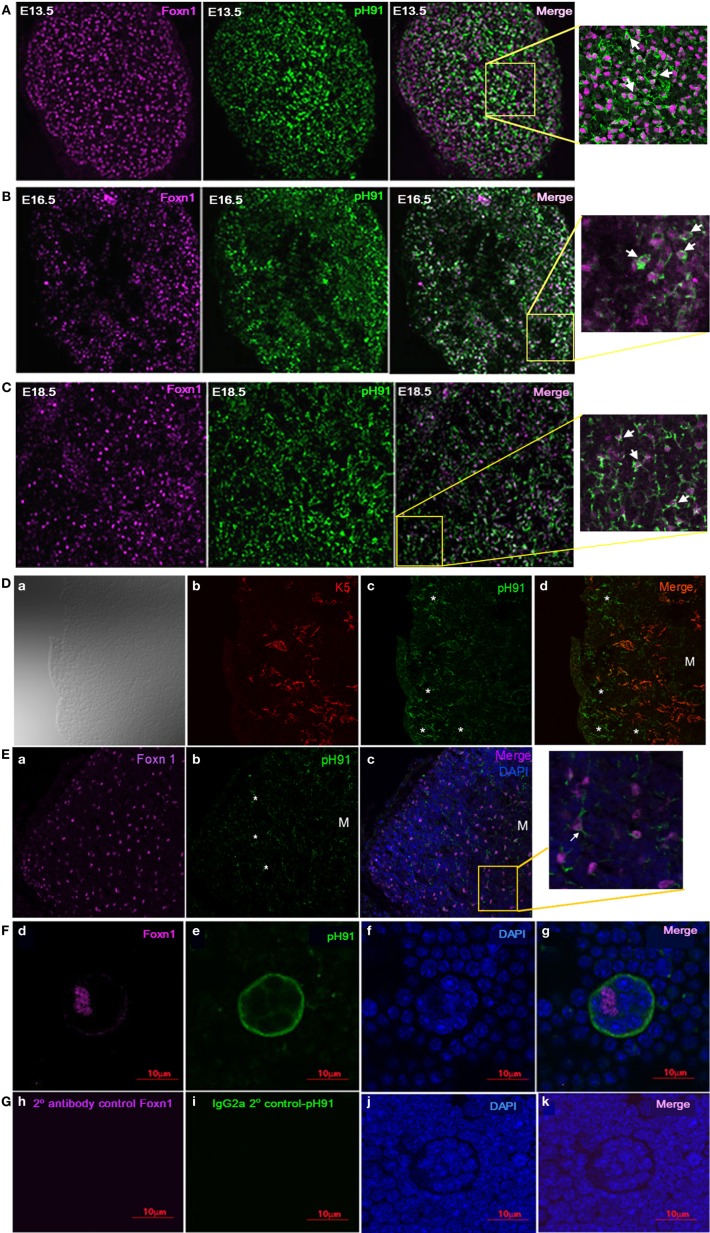
**Expression of Foxn1 and the pH91-antigen in embryonic sections**. Thymic section at embryonic stage E13.5 **(A)**, E16.5 **(B)**, and E18.5 **(C)** were stained with anti-Foxn1 antibody (magenta) and pH91mAb (green). The merged images and insets (**A–C)**, E13.5; E16.5; and E18.5 show an association of pH91^+^ cells with Foxn1^+^ cells. Magnification 20×. **(D)** shows thymic section co-stained with K5 (red) and pH91 (green), The asterisks highlight pH91^+^ cells within the thymic cortex. M indicates the location of the medulla. Magnification 40×. **(Ea–c)** shows the expression of Foxn1 and pH91 in thymic tissue section 2 weeks after birth. Sections were stained with anti-Foxn1 (magenta); pH91 (green). **(Ec)** show merged image and insets of pH91^+^ cells in association with Foxn1 (Arrow). Magnification 40×. **(F)** shows the expression of Foxn1 in pH91^+^ TNC *ex vivo*: Using antibody against Foxn1, expression was observed *ex vivo* in the TNCs. Foxn1 nuclear localization is seen only within TNC nucleus **(Fd)**. pH91 staining is detected **(Fe)**, DAPI nuclear staining **(Ff)** and the merged image is represented in **(Fg)**. Thymocytes do not show Foxn1 expression. **(G)** shows secondary antibody controls for Foxn1 **(Gh)** and pH91 **(Gi)**, All images represent 3 independent experiments. Magnification 40×.

Previous studies implicate the expression of the transcription factor Foxn1 to be required for development of epithelial cells in the thymus (Gordon et al., 2001; Popa et al., 2007). We sought to investigate the relationship between the TNC-antigen, pH91 and Foxn1 during thymic development (Figure 2). The concomitant expression of pH91 with Foxn1 is detectable in cells of the thymic-parathyroid primordium at E11.5 (Figure 2A; inset, lower panel). Similarly, at E12.5 the expression of Foxn1 was also observed with the pH91-antigen throughout the thymic anlagen (Figure 2B). The expression of Foxn1 at this stage of development is consistent with previously published data (Gordon et al., 2001). When we enumerated and compared the phenotypic subtypes of cells present at E11.5 or E12.5, we found cells exhibiting the pH91^−^/Foxn1^+^ phenotype to be significantly represented (78.5%) when compared to the other subsets (Figure 2C). However, by E12.5 we observed a reduction the pH91^−^/Foxn1^+^ phenotype, while cells expressing the pH91^+^/Foxn1^+^ phenotype show a significant increase (Figure 2C). We then examined the ontogenic expression of both Foxn1 and the pH91-antigen in thymic sections obtained at E13.5 through 2 weeks of age. At E13.5 stage of development, Foxn1 and the pH91-antigen expression was detectable (Figure 3A, left and middle panels). The merged image shows the co-localization of both Foxn1 and pH91-antigen in a subset of cells (Figure 3A, right panel). Similar expression patterns for Foxn1 and pH91-antigen were observed at E16.5 and E18.5 (Figures 3B,C). Upon examination of the postnatal thymus (Figures 3D,E), we observed an association of Foxn1 and pH91-antigen. Foxn1+ cells are visible throughout the thymic cortex as well as within the medullary islet (Figure 3E and inset). The presence of pH91^+^ cells are primarily seen in the cortex (Figures 3D,E) and in association with Foxn1^+^ cells (Figure 3Ec and inset). Finally, we examined TNCs *ex vivo* for the presence of Foxn1 and pH91-antigen, we found the Foxn1 transcription factor localized within the nucleus of pH91^+^ TNCs (Figures 3Fd–g).

### Co-expression of Foxn1 and p63 in pH91^+^ TEC

**Figure 4:**
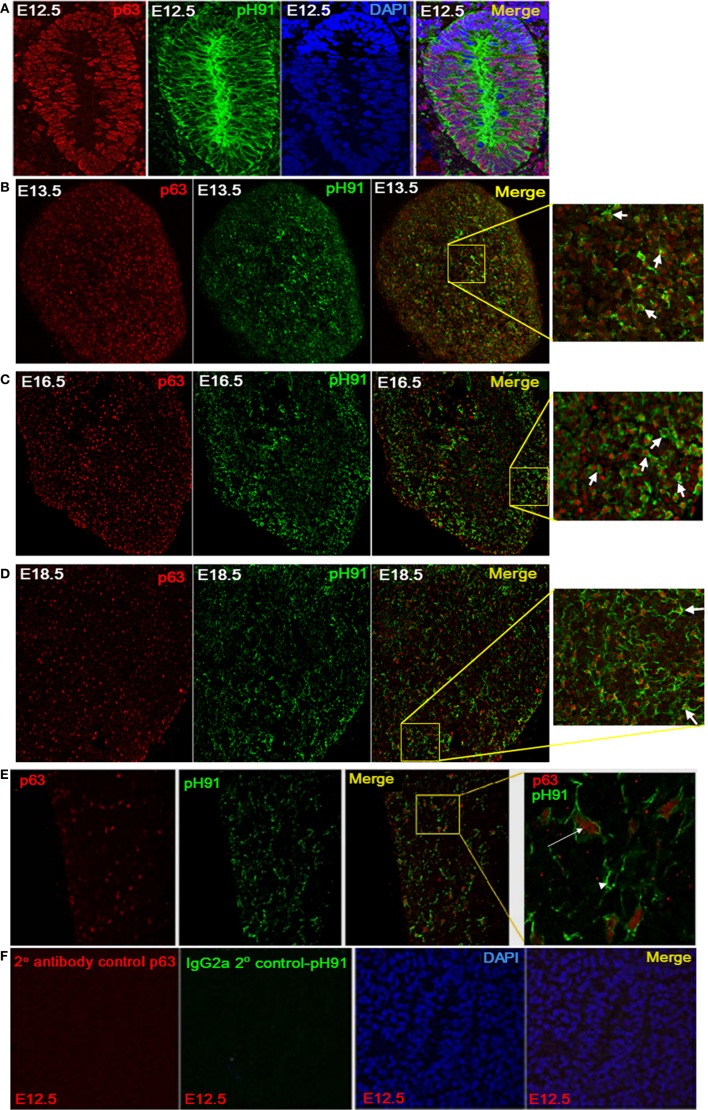
**Expression of p63 by pH91^+^ TECs. (A)** shows thymic anlagen at E12.5 stage of development (sagittal section). Sections were stained with antibodies against p63 (red), pH91 (green), and DAPI (blue). Merged images at each developmental stage are shown in the right subpanels. Expression of p63 is seen in the nucleus of pH91^+^ epithelial cells. **(B–E)** show stained thymic sections with anti-p63 (red) and pH91 (green) at embryonic stages E13.5, E16.5, E18.5 and 2 weeks after birth respectively. Merged images and inset **(B–D)** show pH91^+^ cell expressing p63 (arrow). **(E)** shows pH91^+^ cell expressing p63 (arrow) and pH91^+^ cells that do not express p63 (arrow head). **(F)** shows the secondary antibody and isotype control for p63 (red) and pH91 (green) respectively.

**Figure 5:**
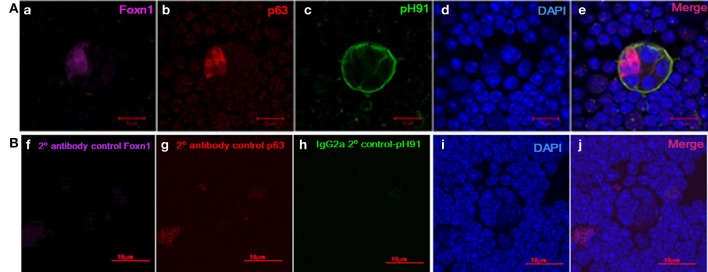
**Expression of Foxn1 and p63 in freshly isolated pH91^+^ multicellular TNCs *ex vivo*. (Aa)** shows *ex vivo* TNC stained with anti-Foxn1 antibody. **(Ab)** shows *ex vivo* TNC stained with anti-p63 antibody. **(Ac)** shows *ex vivo* TNC stained with pH91 mAb antibody. **(Ad)** DAPI stained nuclei. **(Ae)** shows merged image of all three stains. **(Bf–j)** represents antibody controls for Foxn1, p63, and pH91. Magnifications 40×.

The downstream targets of p63 and Foxn1 are not completely known. However, both transcription factors are required for maintenance and growth of the thymus (Gray et al., 2006; Senoo et al., 2007). We examined embryonic tissue and isolated TNCs for the expression of both transcription factors. At E12.5 stage of thymic development, p63 expression is readily detected along with the pH91-antigen (Figure 4A). The expression of p63 persisted throughout embryonic developmental (Figures 4B–D; E13.5, E16.5, and E18.5) and continued expression was observed in the postnatal thymus (Figure 4E). The inset of the merged image shows *in situ* TNCs with nuclear localized p63 (Figure 4E, right panel). E12.5 sections stained with secondary antibody to p63 or pH91 shows no detectable expression of either marker (Figure 4F). DAPI stained nuclei are clearly visible in panels (Figure 4F, 3rd panel and merge). When pH91^+^TNCs were co-stained with both Foxn1 and p63, we detected the expression of these two transcription factors within the nuclei of the cells examined (Figures 5Aa–e).

**Figure 6:**
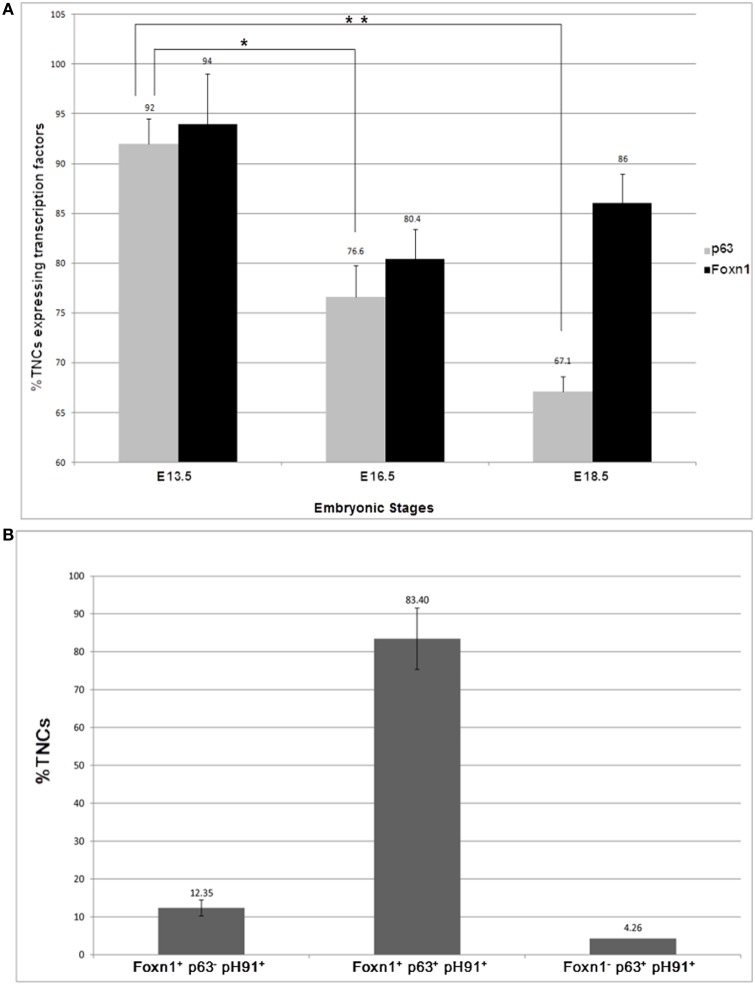
**Quantification of pH91^+^ multicellular TNCs expressing the transcription factors Foxn1 and p63**. pH91^+^ multicellular TNCs were isolated from, thymi from C57BL/6 mice at embryonic stages of development E13.5, E16.5, and E18.5, cyto-spun onto slide and quantified manually with respect to expression of Foxn1, p63 and pH91. **(A)** shows the embryonic expression of p63 and Foxn1 by pH91^+^ cells. **(B)** shows expression of p53 and Foxn1 by freshly isolated TNCs *ex vivo*. These data represent the results (mean ± standard deviation) collected from three independent experiments with three animals per group (*n* = 9). ^*^P < 0.05 compared E13.5 with E16.5. ^**^P < 0.01 compared E13.5 and E18.5. Statistical significance (*t*-test).

To determine the frequency of pH91^+^TNCs that express Foxn1 and p63, we manually counted the number of TNC-complexes that expressed both transcription factors in embryonic and adult mice (Figures 6A,B). During the early stages of development, by E 13.5, there is no significant difference between the expression of Foxn1 and p63 (94 and 92% respectively) (Figure 6A). An evaluation of samples obtained at stage E16. 5 and E18.5 show significant decrease in p63 expression (Figure 5A). Conversely, the Foxn1 expression decreased significantly from E13.5 to E16.5 but rebounded from E16.5 to E18.5 (80.4–86%) (Figure 6A). In neonatal mice, approximately 83% of pH91^+^TNCs were found to express both Foxn1 and p63 whereas, 12.35 % of TNCs were found to be Foxn1^+^ pH91^+^ p63^−^ (Figure 6B).

### The expression of pH91-antigen in the developmental appearance of the TNC multicellular complexes

**Figure 7:**
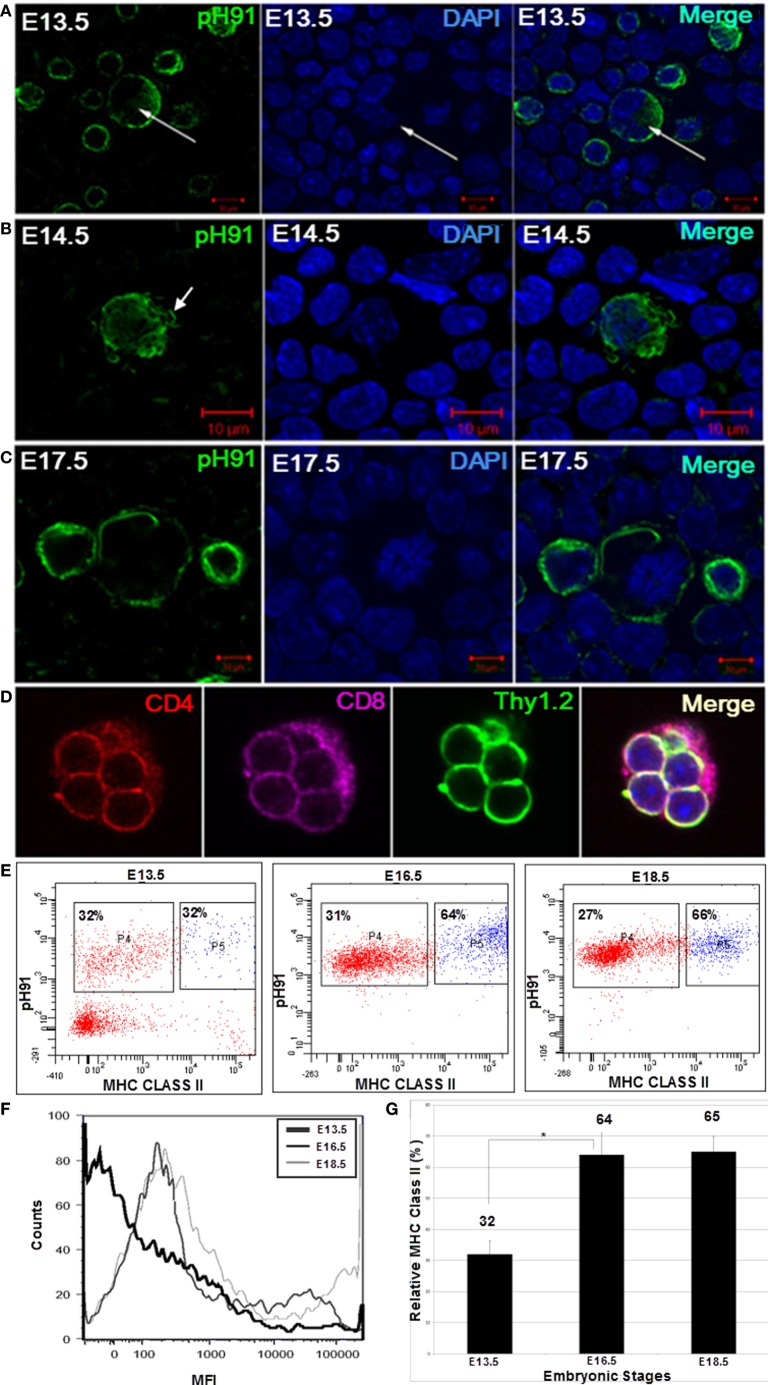
**Ontogeny expression of MHC Class II on the cell surfaces of multicellular TNCs**. The development of TNCs was followed embryonically beginning at E13.5. TNCs were isolated from stages E13.5, E14.5, and E17.5. Isolated cells were cyto-spun onto glass slides and stained with pH91 antibody (green) and DAPI (blue), pH91^+^ cells were seen at different stages of cell cycle. **(A)** shows bi-nucleated cells (arrows). **(B)** shows TNCs expressing fibrous extensions (arrow). **(C)** shows pH91^+^ cells undergoing division. **(D)** shows a small TNC complex isolated from an E17.5 embryo. The TNC is seen with internalized CD4^+^CD8^+^Thy1.2^+^ thymocytes. **(E)** shows FACS analysis for the expression of pH91 and MHC class II by isolated multicellular pH91^+^ TNCs at embryonic stages E13.5, E16.5, and E18.5. **(F)** shows the relative expression of MHC class II by pH91^+^ cells as a function of mean fluorescence intensity (MFI); E13.5 (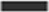); E16.5 (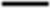); and E18.5 (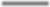). **(G)** shows a statistical significance of MHC expression between E13.5 and E16.5 as well as between E13.5 and E18.5 (^*^p-value < 0.05). Data are representative of three repeated experiments. **(A–C)** Magnification 40×. **(D)** Magnification 63×.

At E13.5 we isolated TECs from embryonic thymi by enzymatic digestion. The resulting cells were visualized microscopically after staining with pH91 antibody. Although pH91^+^ cells were detectable, multicellular complexes were not evident at developmental stages E13.5 and E14.5 (Figures 7A,B). Cells positive for the pH91 antibody during development displayed different phenotypes. Some of the cells were bi-nucleated suggesting cellular division (Figure 7A, arrow), while others showed fibrous extensions (Figure 7B, arrow), characteristic of TNCs isolated from adult mice. The first multicellular complexes become visible at E17.5 (Figure 7C). All of the cells expressed the pH91 antigen. These complexes contained CD4^+^ CD8^+^ double positive thymocytes (Figure 7D). Developmentally, we then examined the time-line for the expression of MHC class II on the surface of these pH91^+^ cells (Figure 7E). At the E13.5 stage, approximately 32% of the total population of cells analyzed was pH91^+^ MHC class II^low^ whereas 32% of the total population of cells analyzed was pH91^+^ MHC class II^hi^. By stage E16.5 pH91^+^ cells show increased expression of MHC class II on their surfaces, 31% MHC class II^low^ and 64 % MHC class II^hi^ (Figure 7E). By embryonic stage E18.5 approximately 66% of pH91^+^ cells expressed MHC class II^hi^ levels and about 27% of pH91^+^ cells were MHC class II^low^ (Figures 7E,G). Alternatively, when we compared the relative levels of MHC class II^hi^ expressed at E13.5, E16.5, and E18.5 using mean fluorescence intensity (MFI), we observed relative increases in MHC class II levels, respectively (Figure 7F).

## Discussion

The thymus is an organ that has been shown to have the ability to undergo regeneration upon injury and irradiation (Popa et al., 2007). This finding suggests that the thymus contains a population of epithelial cells that maintains progenitor function and this function has been shown to persist throughout adulthood and maintain the ability to proliferate and differentiate (Gray et al., 2006). Popa et al. have also shown that K8^+^K5^+^ epithelial cells have the potential to differentiate into mature TECs as well (Popa et al., 2007). Their studies also suggested that K5^+^K8^+^ thymic epithelial progenitors reside in the CMJ (Popa et al., 2007). In a previous publication (Hendrix et al., 2010), we showed that a subset of TNCs are indeed double positive for K5 and K8 cytokeratins, and are strategically positioned within the CMJ of the thymus. The data reported here propose that TNCs have the characteristics of cells previously shown to proliferate and differentiate into mature cortical and medullary TECs.

To explore progenitor potential of TNCs, we used the antibody pH91 to verify along with their distinctive multicellular morphology that the cells of interest were in fact TNCs. First, cells isolated from adult animals with the characteristic TNC phenotype were shown to express K5, K8, and pH91 (Figure 1A, Hendrix et al., 2010). However, it must be pointed out that although the antigen to which pH91 binds is co-expressed with K5^+^K8^+^ by E12.5 stage of development (Figure 6A), the multicellular complexes that define TNCs are only identifiable at day E17.5 (Figure 7C). We propose that the pH91-expressing epithelial cells (non-multicellular) in the early embryo become multicellular through the initial entoses event by E17.5. Our proposal is strengthened by data that show early pH91^+^ (non-multicellular) population and the late pH91^+^ (multicellular) subset to have identical phenotypes for K8, K5, Foxn1, and p63.

Data obtained throughout embryonic development and after birth show a large percentage of pH91-expressing cells to co-express the transcription factors p63 and Foxn1 (Figures 6A,B). With regards to p63, these results suggest that pH91-expressing cells have proliferative potential. Foxn1 expression occurs very early in the development of the thymus (Gordon et al., 2001). Foxn1 is an important transcription factor for thymic organogenesis (Gordon et al., 2001), and is expressed by the bi-potent (K5^+^K8^+^) thymic epithelial cells that have been reported to give rise to mature cTECs or mTECs (Bleul et al., 2006; Senoo et al., 2007; Corbeaux et al., 2010). Further, the expression of Foxn1 is indispensable for attracting lymphoid progenitors into the thymic anlagen and for the cross-talk initiated differentiation of thymic epithelial cells (Gordon et al., 2001; Manley and Condie, 2010).

Foxn1 was first observed in the thymic-parathyroid primordium around E11.25 in a small subset of epithelial cells, with increased expression by E11.75 (Gordon et al., 2001; Manley and Condie, 2010). The expression of Foxn1 was observed in the nucleus of pH91^+^cells in the thymic primordium on day E11.5 (Figure 2), Foxn1 positive only cells were also observed. The expression of the pH91-antigen throughout the thymic-parathyroid primordium suggests that it may be a marker for cells that give rise to both the para-thyroid and thymus. However, the pH91 antigen as well as Foxn1 and p63 only separates with thymic tissue destined to become thymus and is undetectable within the parathyroid (Figure 2). Developmentally, the cytoplasmic membrane expression of the pH91-antigen initiates at about the same time as the nuclear expression of Foxn1. The association of pH91 and Foxn1 is observed throughout the neonatal stages of development (Figures 2, 3, 5, 6). This observation is important because Foxn1 expression is not only required for thymic epithelial cell specification but also critical to postnatal thymic function (Gray et al., 2006; Chen et al., 2009; Cheng et al., 2010). This notion is supported by the findings of Corbeaux et al. (2010) that showed only Foxn1 positive cells are capable of promoting thymopoiesis in the postnatal thymus. Our data show that these same cells express pH91.

The lympho-epithelial structure that defines TNCs was first described in 1980 (Wekerle and Ketelsen, 1980; Wekerle et al., 1980). TNCs were shown to express major compatibility complex (MHC) class I and class II on their cell surfaces (Wekerle and Ketelsen, 1980) as well as surfaces enclosing thymocytes believed to undergoing MHC-restriction. The expression of class II MHC antigen by TNCs along with the autoimmune regulator (Aire) and tissue-restricted antigens (TRAs) provided strong support to the idea that TNCs participate in both positive and negative thymocyte selection (Wekerle and Ketelsen, 1980; Samms et al., 1999; Martinez et al., 2007; Hansenne et al., 2009). The experiments performed here revealed that pH91^+^ cells began expressing high levels of class II MHC in the embryo shortly after internalizing triple positive thymocytes for the first time (66%) (Figures 7E,F). These data suggest that the mouse thymus is competent for MHC restriction by E17.5 (Figure 7D). Collectively, our data suggest that throughout ontogeny, pH91^+^ epithelial cells co-express those antigens associated with thymic epithelial progenitors. These same cells internalize developing thymocytes to form the multicellular complexes that distinguish TNCs from other cell types of the mouse thymus, and soon after this initial entoses event, begin to express high levels of MHC class II antigen which is required for MHC restriction.

### Conflict of interest statement

The authors declare that the research was conducted in the absence of any commercial or financial relationships that could be construed as a potential conflict of interest.
